# Psychophysical predictors of experimental muscle pain intensity following fatiguing calf exercise

**DOI:** 10.1371/journal.pone.0253945

**Published:** 2021-07-30

**Authors:** Nadja Strandberg Kristensen, Emma Hertel, Camilla Hoffmeyer Skadhauge, Sissel Højsted Kronborg, Kristian Kjær Petersen, Megan E. McPhee

**Affiliations:** 1 Faculty of Medicine, Aalborg University, Aalborg, Denmark; 2 Center for Neuroplasticity and Pain (CNAP), Department of Health Science and Technology, Aalborg University, Aalborg, Denmark; Indiana University-Purdue University Indianapolis, UNITED STATES

## Abstract

Musculoskeletal pain affects approximately 20% of the population worldwide and represents one of the leading causes of global disability. As yet, precise mechanisms underlying the development of musculoskeletal pain and transition to chronicity remain unclear, though individual factors such as sleep quality, physical activity, affective state, pain catastrophizing and psychophysical pain sensitivity have all been suggested to be involved. This study aimed to investigate whether factors at baseline could predict musculoskeletal pain intensity to an experimental delayed onset of muscle soreness (DOMS) pain model. Demographics, physical activity, pain catastrophizing, affective state, sleep quality, isometric force production, temporal summation of pain, and psychophysical pain sensitivity using handheld and cuff algometry were assessed at baseline (Day-0) and two days after (Day-2) in 28 healthy participants. DOMS was induced on Day-0 by completing eccentric calf raises on the non-dominant leg to fatigue. On Day-2, participants rated pain on muscle contraction (visual analogue scale, VAS, 0-10cm) and function (Likert scale, 0–6). DOMS resulted in non-dominant calf pain at Day-2 (3.0±2.3cm), with significantly reduced isometric force production (P<0.043) and handheld pressure pain thresholds (P<0.010) at Day-2 compared to Day-0. Linear regression models using backward selection predicted from 39.3% (P<0.003) of VAS to 57.7% (P<0.001) of Likert score variation in DOMS pain intensity and consistently included cuff pressure pain tolerance threshold (P<0.01), temporal summation of pain (P<0.04), and age (P<0.02) as independent predictive factors. The findings indicate that age, psychological and central pain mechanistic factors are consistently associated with pain following acute muscle injury.

## Introduction

Musculoskeletal pain is estimated to affect approximately 20% of individuals globally [1]. Mechanisms underlying the transition from acute to chronic pain are not fully elucidated. However, demographic factors, such as age and sex [2–9], together with other individual lifestyle and psychosocial factors, and alterations in pain processing mechanisms may contribute to the risk of chronic pain development.

Regarding lifestyle and psychosocial factors, sleep, physical activity, affect and pain-related catastrophizing seem to play an important role in pain development and/or exacerbation. For example, epidemiological studies have demonstrated associations between poor sleep quality and development of widespread pain [10, 11], and sleep deprivation has been found to inhibit descending pain modulation [12], indicating a potential mechanistic contribution to pain exacerbation. In addition, sedentary lifestyle has been associated with a pro-inflammatory state [13], which has further been associated with e.g., increased risk of higher clinical pain intensities after surgery [14]. In contrast, higher self-reported leisure time physical activity has proven to be predictive of less pain and disability in people with chronic low back pain [15], and is associated with improved central pain processing, such as increased conditioned pain modulation (CPM) and reduced temporal summation of pain (TSP) [16]. Positive affective state has also previously been observed to be associated with decreased pain severity in people with chronic pain [17], while studies have suggested that higher pain catastrophizing is associated with reduced CPM, indicating a less efficient pain inhibitory system [18], and higher levels of clinical pain after e.g., surgery [19] and general practitioner treatment [20].

Measures of pain processing, such as TSP and CPM, have also been heavily researched in recent years as potential mechanistic contributors to variation in pain development and experience. These measures can be investigated using quantitative sensory testing (QST), where different sensory modalities are applied and psychophysical thresholds can be quantified [21, 22]. Pressure-based stimuli are often used to assess musculoskeletal pain [23–25] with an increase in pressure pain sensitivity being indicative of localized mechanical sensitization at the injured or painful site [25]. TSP; a phenomenon in which a sequence of repeated noxious stimuli at frequencies >0.33Hz are applied and resulting in increases in pain perception are recorded; is often used to quantify facilitation of nociceptive transmission [23, 24, 26]. While CPM; a ‘pain-inhibits-pain’ paradigm [27, 28]; is used to capture descending noxious inhibitory capacity [29]. Facilitated TSP and reduced CPM have previously been associated with higher pain intensity after surgery [23, 30, 31] and baseline TSP has previously been suggested to predict peak exercise-induced pain intensity [24].

Individual QST measures and other factors have previously been investigated as possible predictors of exercise-induced pain, or delayed onset muscle soreness (DOMS) [24, 32–35]. DOMS mimics aspects of mild musculoskeletal pain [24, 36, 37], peaking 48 hours after intensive or unfamiliar exercise [36, 38], with the sensation varying from slight stiffness to severe pain with restrictions in movement [39]. As DOMS can be evoked under standardized conditions, this approach offers the unique possibility to assess potential predictors in a pain-free state, and then relate them to subsequent pain development of DOMS.

This study aimed to assess a range of known predictors from people with chronic pain and to utilize these in a predictive model for the acute onset of pain following DOMS in healthy subjects.

## Methods

**Fig 1:**
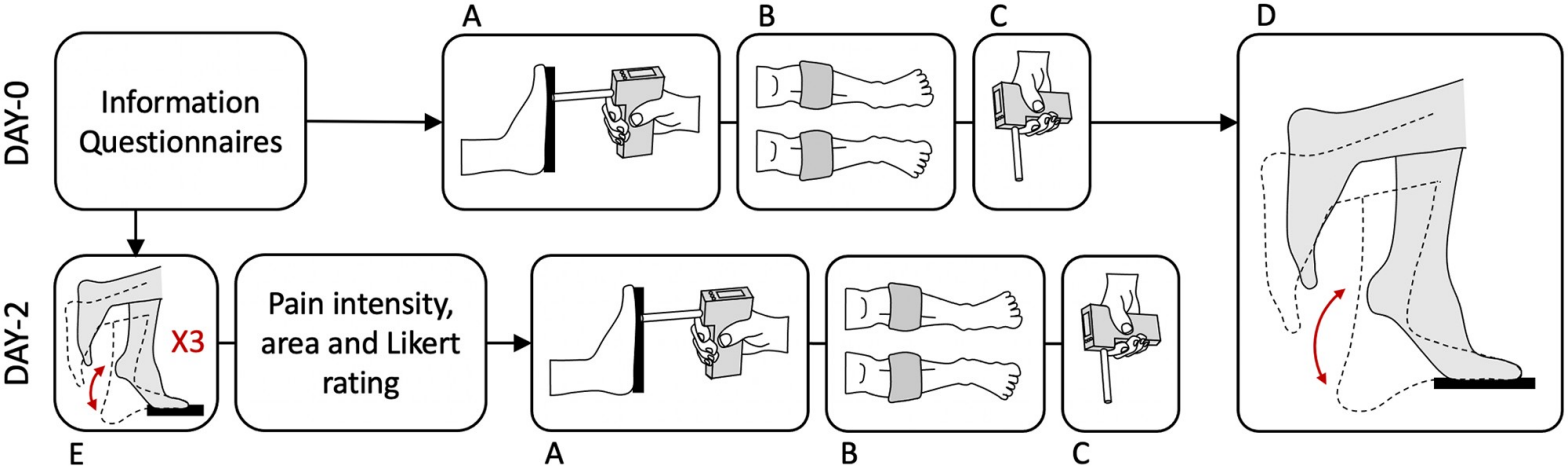
Overview of protocol showing timeline on Day-0 and Day-2 including: Initial information, questionnaires and ratings on Day-0, A—isometric force measurement, B—cuff algometry measurement of pain sensitivity, C—handheld pressure algometry measurement of pain sensitivity, D—delayed onset muscle soreness induction by calf raises, and E—assessment of pain intensity and area on contraction following 3 calf raises.

Two experimental sessions were scheduled for each participant, separated by 48 hours. On Day-0, participants answered questionnaires about demographics, physical activity, pain catastrophizing, affective state, and sleep quality. This was immediately followed by measurement of isometric plantar flexion force production. Computer-controlled cuff-pressure algometry was used to assess cuff pain detection and tolerance thresholds, TSP and CPM, and handheld pressure algometry was used to assess local, contralateral and remote pressure pain thresholds. Participants then performed repeated calf raises until fatigue to evoke DOMS. On Day-2, questionnaires were followed by three calf raises with subsequent rating of pain intensity and drawing pain area, before isometric force measurement and pain pressure sensitivity assessments with both cuff and handheld algometry were completed as on Day-0 (Fig 1). The protocol was approved by the local ethical committee (N-20180089) and was conducted at Aalborg University, Denmark, in November-December 2020.

### Participants

Healthy participants between the age of 18–45 were recruited from Aalborg University, Aalborg, Denmark and wider community. Individuals were excluded if they reported: drug addiction; previous or current neurological or musculoskeletal conditions, mental illness, pulmonary, cardiac or chronic pain conditions; lack of cooperation; current use of medications with potential effect on the trial (e.g., analgesics or anti-inflammatory drugs); consumption of stimulants or painkillers on the day of the experiment; or recent history of pain. Furthermore, individuals who frequently (>2 times/week) did weighted calf exercises were excluded. Eligible participants were provided with verbal and written information and gave written informed consent before the first session.

### Questionnaires

A demographic questionnaire was verbally administered by the investigator on Day-0 and included information regarding age, weight, height and leg dominance. Four validated questionnaires were then completed by the participants in the following order: International Physical Activity Questionnaire: Short Form (IPAQ) [40] to capture physical activity and score as metabolic equivalent of task (MET) minutes per week; Pain Catastrophizing Scale (PCS) [41] to capture pain-related thoughts and distress; Positive and Negative Affective Schedule (PANAS) [42] to capture current affective state; and Pittsburgh Sleep Quality Index (PSQI) [43] to capture sleep quality.

### Experimental muscle pain induction

To induce experimental muscle pain, a DOMS protocol was used in which unilateral calf raises on the non-dominant (index) leg were completed to fatigue, as previously utilized in other studies [36, 44]. Participants stood on a step on the ball of their non-dominant foot with the heel hanging off the edge, keeping their dominant foot lifted throughout (Fig 1D). Participants were instructed to raise and lower the heel through full range of motion, performing the eccentric phase in a slow controlled manner over three to five seconds. Participants were allowed to place their fingertips on the railing for balance. The exercise was terminated by the investigator when participants could no longer reach full range of motion or maintain the correct tempo for three consecutive calf raises [45].

#### Assessment of experimental muscle pain

Pain during muscle contraction was assessed on Day-2, immediately following performance of three calf raises, using a visual analogue scale (VAS, anchored at 0 cm: no pain, and 10 cm: worst pain imaginable) for pain intensity and paper body chart for pain area. In addition, a 7-point Likert scale of muscle pain for lower limb was used (0, a complete absence of pain; 1, a light pain felt only when touched/a vague ache; 2, a moderate pain felt only when touched/a slight persistent pain; 3, a light pain when walking up or down stairs; 4, a light pain when walking on a flat surface/painful; 5, a moderate pain, stiffness or weakness when walking/very painful; and 6, a severe pain that limits my ability to move) was used to measure pain interference in functional tasks.

### Assessment of isometric plantarflexion force

A handheld algometer modified with a footplate was used to assess muscle force production by measuring isometric plantarflexion force. This method was chosen to allow direct assessment of maximal voluntary isometric contractions of the index gastrocnemius muscle to validate the DOMS model and assess predictive value of the muscle strength. Based on within-session repetitions from the present study, the method is reliable session 1 (Interclass correlation coefficient (ICC) = 0.876, P<0.001) and session 2 (ICC = 0.849, P<0.001). In reclined long sitting with the foot in plantigrade on the footplate, participants were instructed to produce maximal plantarflexion force by pushing their forefoot into the footplate without lifting the heel. The handheld device and footplate were stabilized manually by the examiner during the assessment. This was repeated three times per participant in each session. Measurements were pooled and averaged for each participant.

### Computer-controlled cuff pressure algometry

To assess pressure pain sensitivity, a computer-controlled cuff pressure algometer (Cortex Technology, Aalborg, Denmark) paired with two 13 cm air cuffs (VBM Medical, Sulz a. N., Germany) and an electronic VAS (eVAS, anchored as per VAS above) (Cortex Technology, Aalborg University, Denmark) was used [25]. The cuffs were placed over the widest portion of both calves, approximately 10 cm below the tibial tuberosity.

#### Cuff pain detection and tolerance threshold

A ramped test with a constant cuff inflation rate of 1 kPa/s and a safety-cap of 100 kPa was used to determine cuff pain detection threshold (cPDT), cuff pain tolerance threshold (cPTT), and cuff pain tolerance limit (cPTL) on each leg; first on the non-dominant (index) lower leg, then on the dominant (contralateral) lower leg. Participants were instructed to start moving the dial on the eVAS when the pressure first became painful, continuously rating the pain throughout the ramp, and pressing the stop button when they could not tolerate further increases in pressure. The cPDT was defined as the pressure (kPa) when the eVAS reached 1 cm, as used in previous studies [46, 47]. cPTT was defined as the pressure (kPa) when the participant pressed the stop button. cPDT, and cPTT were each averaged across both legs at Day-0 for linear regression analysis.

#### Temporal summation of pain

For assessment of TSP, ten consecutive pressure stimuli (1s ON; 1s OFF) were applied to the index lower leg (i.e. the leg in which DOMS was induced) at cPTT intensity. Participants were instructed to rate the intensity of pain from the first inflation on the eVAS and then adjust for the following inflations without returning the eVAS to zero. For each inflation, an eVAS score was extracted, and TSP was calculated as the difference between the averaged eVAS score for the last three stimuli and the first four stimuli, as used in previous studies [48, 49].

#### Conditioned pain modulation

For assessment of CPM, the test stimulus was a ramped inflation on the index lower leg at a rate of 1 kPa/s and a safety-cap at 100 kPa, as above, simultaneously with a constant conditioning stimulus at 70% cPTT on the contralateral leg. The CPM-effect was calculated by subtracting the cPDT measured during conditioning from the cPDT recorded during the first ramp [50].

### Handheld pressure algometry

Pressure pain threshold (PPT) was assessed at three different body sites using a handheld pressure algometer (Somedic, Solna, Sweden) with a 1 cm flat probe [51]. All sites were marked to ensure that pressure was applied at the same spot for each repetition. The sites were: bilateral gastrocnemius muscles (midpoints measured between the calcaneus and the popliteal line) and the right upper trapezius muscle (midpoint measured between the seventh cervical spinous process and the acromion tip). The probe was placed perpendicular to the skin and pressure was applied at a rate of 30 kPa/s. Participants were instructed to indicate when the pressure first became painful by pressing a button. Pressure was applied three times at each site in a rotating schedule, starting at the left gastrocnemius muscle, continuing to the right gastrocnemius muscle, and ending at the upper right trapezius, with approximately one-minute break between each repeated measure. Measurements were averaged for each site for analysis.

### Statistical analysis

Using G*Power, an A-priori sample size calculation, based on a prior linear regression model for DOMS intensity prediction (R^2^ = 28.6% [24]) with effect size of 0.35 [24], alpha level of 0.05, power of 0.8, and 1 expected independent predictor [24], deemed 25 participants necessary to achieve statistical power. Hence, 30 participants were recruited to account for dropouts and non-responders to DOMS induction.

Parametric data are presented as mean (± standard deviation, SD) and non-parametric data are presented as median (interquartile range, IQR). For investigation of the primary outcome, a linear regression analysis with backward selection was used to determine whether baseline parameters could be used as predictors for the intensity of muscle pain on Day-2. Assumptions (linearity, independent residuals, homoscedasticity, no multicollinearity, normality, and no outliers) were all assessed with corresponding statistical or visual methods. Pain intensity (VAS) on muscle contraction on Day-2 was defined as the dependent variable with independent variables including age, sex, BMI, baseline positive and negative PANAS scores, PSQI, IPAQ, PCS, isometric force, handheld PPTs, mean cPDT, mean cPTT, TSP, CPM, and number of exercise repetitions. A similar regression analysis was run with Likert-scale score as dependent variable being the only change. For investigation of changes in psychophysical measures, all data were checked for normality using the Shapiro-Wilks test, and corresponding paired-samples t-test or Wilcoxon signed-rank test were used to compare changes in isometric muscle force and pain sensitivity (PPTs, cPTT, cPDT, TSP, CPM) between days (Day-0/Day-2). Two-way mixed ICCs with absolute agreement were used to determine within session reliability of the modified footplate. Statistical analysis was performed in SPSS statistics (IBM SPSS Statistics for Windows, Version 27.0) and significance was accepted at P<0.05.

## Results

### Participant characteristics

Thirty participants (15 female) were recruited. Two participants did not complete the second experimental day, due to migraine and varicella-infection, and were excluded. The remaining 28 participants (13 female) all completed both sessions and were included in the analysis. All participants reported being right leg dominant. Full sample characteristics and questionnaire data can be seen in Table 1, with anonymized individual data for all major outcomes available in the (S1 Data).

**Table 1:** Participant characteristics and questionnaire responses on Day-0.

Characteristic	Mean ± SD or Median (IQR)
**Age**	22.5 (3) years
**BMI**	22.7 ± 2.5 kg/m^2^
**Sex**	15 males: 13 females
**Sleep**	8 (1) hours
**PCS**	14.6 ± 7.0
**PANAS-Neg**	12 (1)
**PANAS-Pos**	31 (7.5)
**PSQI**	4 (2.25)
**IPAQ**	2453 ± 1528 MET-min/week

BMI, body mass index; PANAS; Positive and Negative Affective Schedule (Pos = Positive subscale, Neg = Negative subscale); PSQI, Pittsburgh Sleep Quality Index; IPAQ, International Physical Activity Questionnaire; MET-min/week, metabolic equivalent task minutes per week.

### Induced delayed onset muscle soreness of index gastrocnemius muscle

**Fig 2:**
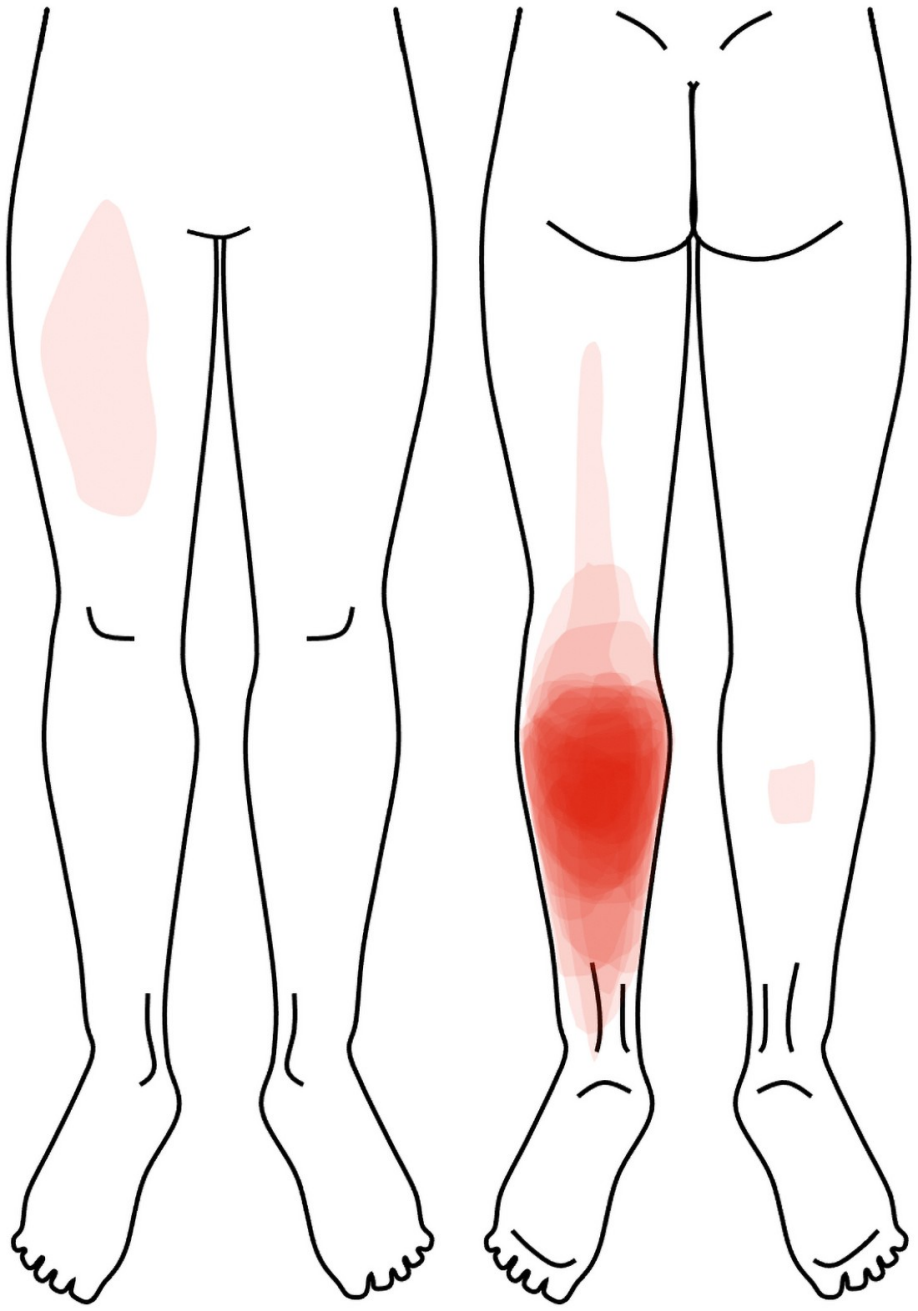
Overlay of all participants location of pain during calf raises on Day-2.

Pain intensity (VAS) during calf raises on Day-2 was reported to be 3.0 ± 2.3 cm, with median muscle soreness on the Likert scale being 4 (2.25) corresponding to “a light pain when walking on a flat surface/painful”. As shown in Fig 2, all participants indicated pain over the non-dominant calf (DOMS induced leg), with three participants also marking other areas (i.e. ipsilateral posterior thigh, contralateral anterior thigh, and contralateral calf).

### Effects of experimental muscle pain on force production and pain sensitivity

All force and pain sensitivity data from Day-0 and Day-2 can be seen in Table 2. Mean force was reduced on Day-2 compared to baseline (Z = -2.02, P<0.043), as was handheld PPT over the index gastrocnemius (t_27_ = 2.75, P<0.010). No significant changes were observed in cPDT, or cPTT for either leg. Similarly, no significant differences were found for TSP or CPM, nor for PPT at the contralateral leg or upper trapezius sites.

**Table 2:** Isometric force production and pain sensitivity data at Day-0 and Day-2.

Outcome (kPa)	Baseline (Day-0)	During DOMS (Day-2)
Isometric force	718 (575)	544 (487)*
cPDT (index)	26.2 (18.5)	29.5 (16.8)
cPDT (contralateral)	29.2 (14.2)	28.4 (9.0)
cPTT (index)	73.2 (27.2)	71.6 (40.2)
cPTT (contralateral)	67.9 (32.2)	68.6 (39.7)
TSP	1.3 ± 1.6	1.8 ± 1.3
CPM (ΔPDT)	9.6 ± 12.5	5.5 ± 12.5
PPT Gas. (index)	402.8 ± 186.7	355.0 ± 172.2*
PPT Gas. (contralateral)	387.9 ± 179.2	370.8 ± 157.5
PPT Upper Trapezius	303.0 (187.5)	279.0 (179.8)

Values are presented as mean ± standard deviation or median (interquartile range). cPDT, cuff pain detection threshold; cPTT, cuff pain tolerance threshold; TSP, temporal summation of pain; CPM, conditioned pain modulation; PPT, pressure pain threshold; Gas., Gastrocnemius muscle. Significant difference between days indicated (*, P<0.05)

### Predicting muscle pain intensity using baseline variables

All assumptions for conducting a multiple linear regression model with backward selection were met, with contralateral and upper trapezius PPTs excluded due to high collinearity with index PPTs. Therefore, the initial model included Day-0 assessments of age, sex, BMI, number of calf raise repetitions, questionnaire scores (positive and negative PANAS, PSQI, IPAQ, and PCS), isometric force, and pain sensitivity variables (index gastrocnemius PPT, mean cPDT, mean cPTT, TSP, CPM) and was non-significant with an adjusted R^2^ of 23.7% (F_15,12_ = 1.56, P>0.22, Table 3). Following 12 iterations, removing the least significant variable at each iteration, the final model significantly explained 48.3% of variance with an adjusted R^2^ of 39.3% (F_4,23_ = 5.36, P<0.003), and included the following independent predictor variables: age, positive PANAS score, TSP, and mean cPTT.

**Table 3:** Initial and final linear regression model statistics for prediction of Day-2 pain ratings.

Initial Model					
	B	Standard error	Beta	t	Sig.
**Constant**	10.97	7.23		1.52	0.16
**Age**	-0.38	0.21	-0.45	-1.85	0.09
**Sex**	1.05	1.40	0.24	0.75	0.47
**BMI**	-0.01	0.18	-0.01	-0.05	0.96
**# Repetitions**	0.03	0.06	0.12	0.45	0.66
**PANAS-Pos**	0.17	0.11	0.41	1.54	0.15
**PANAS-Neg**	-0.29	0.32	-0.23	-0.91	0.38
**PSQI**	0.12	0.28	0.10	0.42	0.68
**IPAQ**	0.00	0.00	0.19	0.96	0.36
**PCS**	0.01	0.09	0.04	0.13	0.90
**Isometric force**	0.00	0.00	-0.08	-0.38	0.71
**PPT index Gas.**	0.00	0.00	-0.18	-0.65	0.53
**Mean cPDT**	0.00	0.06	-0.01	-0.02	0.98
**Mean cPTT**	-0.06	0.04	-0.46	-1.25	0.24
**TSP**	0.61	0.28	0.43	2.19	0.05*
**CPM**	0.01	0.04	0.07	0.34	0.74
**Final Model**					
**Constant**	9.74	3.90		2.50	0.02*
**Age**	-0.35	0.13	-0.42	-2.63	0.02*
**PANAS-Pos**	0.15	0.07	0.35	2.30	0.03*
**Mean cPTT**	-0.05	0.02	-0.45	-2.88	0.01*
**TSP**	0.61	0.22	0.43	2.79	0.01*

PANAS, Positive and Negative Affective Schedule (Pos = Positive subscale, Neg = Negative subscale); PPT, pressure pain threshold; Gas., Gastrocnemius muscle; TSP, temporal summation of pain; cPTT, cuff pain tolerance threshold; # Repetitions, number of calf raises completed. Significant independent predictive value indicated (P<0.05)

As mean cPTT was truncated in 5 participants, where the safety limit of 100kPa was reached in both legs on Day-0, the regression analysis was re-run to ensure this did not impact the model. In this case, the final model included the same four variables (age, positive PANAS score, TSP and mean cPTT) and remained significant (F_4,18_ = 11.46, P<0.05), explaining 40.6% of the variance with an adjusted R^2^ of 27.3%. In both cases, higher muscle pain intensity at Day-2 was associated with younger age, higher positive affect, lower mean cPTT and increased TSP.

A similar model could also be obtained when the dependent variable was changed to Likert ratings of lower limb muscle soreness, in which the final significant model following 12 iterations explained 64.0% of variance with an adjusted R^2^ of 57.7% (F_4,23_ = 10.20, P<0.001). This final model included significant independent predictor variables of age (P<0.001), calf raise repetitions (P<0.01), mean cPTT (P<0.01) and TSP (P<0.04), whereby younger age, completion of more repetitions, lower mean cPTT and higher TSP were associated with higher Likert scores.

## Discussion

This study aimed to investigate DOMS in the lower leg as an experimental muscle pain model and then identify baseline predictors of muscle pain intensity. The exercise successfully induced DOMS in the current study with presence of pain on contraction of the calf muscle, soreness when walking, reduced muscle force production and local pressure pain hypersensitivity, consistent with prior studies [24, 32, 33]. Age, baseline mean cPTT and baseline TSP were identified as consistently significant explanatory variables for variance in pain and muscle soreness on Day-2. Positive affect and number of repetitions performed also contributed significantly to individual regression models. It seems evident that pain is multimodal and that factors such as central pain mechanisms [23, 26–28, 52], psychophysical factors [10, 11, 16–19], sex [2, 3, 7], and age [3, 4, 6] contribute to the experience of pain. McPhee et al., 2019 demonstrated that a baseline facilitated TSP response was associated with higher pain intensities to a low back pain DOMS model in healthy subjects [24] and this correspond well with the studies demonstrating that pre-treatment TSP is associated with future pain or pain after various treatments [30]. The current study adds to the literature, since this is the first study to demonstrate a predictive value of TSP, positive affect, sex, and age on the pain intensity of DOMS.

### Experimental muscle pain model

Fatiguing calf raise protocols have previously been used to induce DOMS in the triceps surae musculature [36, 44]. Hallmarks of DOMS include pain on movement [24, 34], reduced muscle force production [33, 44, 53] and reduced function [24] all of which were present in this sample on Day-2. Participants in the present work performed a similar number of calf raises to those in prior studies [54], and reported mild pain (between 2-3/10) also consistent with various prior experimental DOMS models [24, 34]. Furthermore, a significant reduction in isometric force was observed on Day-2, which is in line with the typical finding of reduced muscle strength [33, 53] that may last for up to four days following DOMS induction [53]. Heightened pressure pain sensitivity was also observed as demonstrated previously [44], suggesting induction of peripheral sensitization in the exercised muscle as a result of microstructural muscle damage [36]. In combination, these findings suggest DOMS induction was successful in the present work, evoking similar features to prior DOMS models and to mild clinical musculoskeletal pain conditions.

### Changes in pain sensitivity due to experimental pain

Although local PPT was reduced, no changes were observed for PPT at the contralateral leg or upper trapezius, nor when pain thresholds, TSP or CPM were assessed with cuff pressure algometry. This indicates that sensitisation was confined to the region of muscle damage with no clear widespread or central effects as have been reported in prior models with higher pain intensity [34] or using larger muscle groups [24].

Compared to handheld pressure algometry, computer-controlled cuff-pressure algometry evokes pain in a larger volume of tissue with peak strain and stress primarily under the cuff, though, not confined to that area [55, 56]. Cuff pressure algometry can potentially create ischemia when applied at pressures near 250mmHg for several minutes at rest. However, shorter and lower intensity cuff pressure assessment paradigms are not likely to induce ischemic pain, so this is unlikely to explain differences between cuff and handheld algometry results [57]. In contrast, single-point pressure stimulation is characterised by stress distributed close to the pressure-point with no distortion of tissue elsewhere [56]. At pain threshold, computer-controlled cuff-pressure algometry therefore produces a mean strain on the gastrocnemius muscle surface of 0.102 [55] in comparison to a mean strain of 0.210 from handheld pressure algometry [58]. This suggests that cuff thresholds, despite assessing deeper tissues, may be less specific to the muscle affected in the present work and thus less impacted by changes in local sensitivity.

Central pain processing mechanisms were quantified in the current study by assessment of TSP and CPM, neither of which were altered in the presence of DOMS. Whether TSP changes over an episode of DOMS is debated with some studies reporting a difference [34, 35], while others do not [24]. As TSP is theorised to represent segmental hyperexcitability in the dorsal horn [59], the assessment site and modality become critical. Although the assessment in the current study was performed over the painful calf, as indicated above, this technique may not have captured facilitation produced specifically within the segmental innervation of the triceps surae. Unlike TSP, CPM has consistently been shown to be stable over an episode of DOMS [24, 49, 60] and does not seem to be affected by short bouts of mild pain [60]. This may be because pain from DOMS is primarily felt during movement [61], thus, not producing the continuous stimulation thought to be necessary for evoking central changes [26, 49]. The lack of changes in central mechanisms observed here are hence not unexpected and can likely be attributed to the lack of significantly continuous, intense or widespread pain [62] and the short pain duration [63].

### Baseline prediction of experimental muscle pain intensity

The strongest predictor of pain intensity during DOMS was cPTT. Higher baseline tolerance was associated with lower DOMS severity, which is consistent with literature, showing lower pain tolerance thresholds generally to be present in chronic pain populations [64, 65] associated with non-recovery after injury [66] and clinical pain intensity [67], development of persistent post-operative pain [68, 69], various other health-related factors including analgesic consumption [70] and lack of physical activity [71]. Compared to pain detection thresholds, pain tolerance has long been thought to reflect more cognitive-evaluative aspects [72]. It may, therefore, act as an experimental proxy of hypervigilance or pain-related fear [73], which also presumably play a significant role in evaluating muscle pain intensity.

TSP contributed significantly to each model as an independent predictor, with facilitated TSP found to be predictive of higher pain intensity. This is in accordance with literature, finding facilitated TSP to be predictive of higher DOMS pain intensities [24], poor analgesic effects, and higher post-operative pain intensities [23, 30, 74]. TSP is commonly purported to represent a pronociceptive profile, reported to associate with pain hypervigilance and clinical pain intensity, and commonly facilitated in people with both acute and chronic musculoskeletal pain [52, 75, 76]. As with pain tolerance, higher baseline TSP would therefore seem to characterise individuals who show increased vulnerability to pain development.

The current study further identified age to be associated with perception of more severe pain, though this relationship between age and pain has been debated previously [3, 5–9]. A recent systematic review investigating predictors of postoperative pain and analgesic consumption, found younger age to be commonly associated with increased pain intensity and analgesic need after surgery [6], consistent with the association observed here. However, reports of the opposite tendency [8] or no significant association [5, 7, 9] are also common. While the present finding is interesting, it should be noted that all participants were between 19–32 years, thus, the effect of age in a broader sample remains to be investigated.

Prior studies have shown that negative cognitions, such as pain-related catastrophizing and fear of pain, are influential to or predictive of pain intensity in other DOMS models [32, 77]. Likewise, anxiety and depressive symptoms have also been shown to predict loss of range of motion subsequent to DOMS [78]. In contrast, the present study found positive affect to predict experimental pain intensity in some models. For affect, this is consistent with prior suggestions that affect can moderate pain perception [79]. Contrary to the present finding, a recent meta-analysis identified a significant negative association between positive affect and chronic pain severity in observational studies [17]. It also seems more logical to expect that positive affect would be related to reduced pain, as experimental induction of positive mood has been shown to increase activity in brain regions linked with pain modulation and to reduce experimental pain perception [80]. Nevertheless, it is possible that higher positive affect in the present study is a by-product of increased engagement in or nervousness about the experiment, consistent with selection of positive words in the PANAS such as “active”, “attentive”, “alert”, “excited”, “interested” or “enthusiastic”. While this is purely speculation, such a state may actually drive individuals to perform more repetitions relative to their physical capacity than those who were less engaged, leading to higher pain development on Day-2. Further investigation into more nuanced classifications of affect could provide interesting insights into the role of contextual factors and participant engagement in experimental results.

### Perspectives

Experimental models of muscle pain in standardised settings allow for investigation of mechanisms involved in pain development [81], making it possible to investigate pain modulating factors and QST for prediction of pain intensity in a pain-free state. In clinical populations, a pronociceptive profile, defined as low pain thresholds, high TSP, and low CPM, has been found predictive of higher pain intensities [30, 74]. Similarly, the model in the current study identified low cPTT and increased TSP as predictors of pain intensity after experimentally induced DOMS. Such phenotyping of pain conditions might be useful in the prediction of therapeutic response, as both CPM and facilitated TSP are predictive of lowered pain-alleviating effects of treatment [30, 47, 74, 82]. Future studies should continue to evaluate the underlying physiological mechanisms for, and the predictive value of, the variables identified here in an ultimate attempt to develop personalized pain therapy.

### Limitations

The current study used a within-participant design with no control group. Therefore, it was not possible to control for potential habituation effects of repeated assessment [83]. While both the methods used to assess pain sensitivity [25, 49, 51, 84], as well as the questionnaires [40–43], have shown excellent reliability in similar conditions, the predictive value of the model depends solely on how adequately these measures are captured in the current study. For the computer-controlled cuff-pressure algometry mean cPTT was truncated in 5 participants. This may have led to an underestimation of the true predictive value of cPTT for pain intensity.

The Likert scale was analyzed using linear regression, a parametric analytical approach, which has previously been subject to controversy. However, the current study chose the linear regression based on previous reports arguing parametric statistics to be robust in this context [85, 86]. Both linear regression models also used a backward selection approach, which offers the ability to assess combined predictive ability of all entered variables and to reduce the model in a standardized manner to only include the most important variables in the final model. Disadvantageously, dropped variables cannot be re-entered into the model, even though they might be significant in later iterations [87], but in comparison to forward selection or stepwise models, backward selection does not require strict preselection and can better identify useful combinations of predictors without requiring individual explanatory value of each variable. The current study assesses the predictive value of the entirety of the measured psychophysical measures as an individual pain profile, why assessment of joint predictive value of the variables is appropriate. Additionally, the power estimation was based on a prior study identifying only 1 predictor [24]. As the current study identified four predictors of DOMS pain intensity, there might be a risk of model saturation.

## Conclusion

The current study successfully induced DOMS in the non-dominant calf muscle. Baseline reduced mean cPTT, increased positive affect, facilitated TSP, younger age and completion of more exercise repetitions, predicted muscle pain intensity on Day-2. This highlights factors which may be important to investigate in further research to uncover why some individuals develop more severe pain than others following similar musculoskeletal injuries. This has clinical implications, as identification of high-risk individuals might offer options for earlier and/or personalised therapy to prevent increases in pain severity and pain persistence.

## Supporting information

S1 Data Anonymized individual participant data for all major outcomes. (PDF)

S1 Checklist STROBE statement—Checklist of items that should be included in reports of observational studies. (DOCX)
