# Supplementary material for: Psychophysical predictors of experimental muscle pain intensity following fatiguing calf exercise
Source: PLoS One. 2021 Jul 30;16(7):e0253945. doi: 10.1371/journal.pone.0253945 (PMC8323909; doi:10.1371/journal.pone.0253945)
Supplement: S1 Data — (PDF) [file pone.0253945.s001.pdf]

S1 Data: Individual Data for all main outcomes on Day-0, pain reports on Day-2 and change in psychophysical outcomes to Day-2.

Baseline (Day-0) Individual Data for all Main Outcomes														DOMS (Day-2)		Change in Psychophysical Outcomes (Day-2)							
ID	PCS	IPAQ	Neg	Pos	PSQI	Force	cPDT	cPTT	TSP	CPM	PPTi	PPTc	PPTu	Likert	VAS	Force	cPDT	cPTT	TSP	CPM	PPTi	PPTc	PPTu
1	17	960	11	34	10	482	17.4	49.4	2.0	19	534	577	536	5	7.0	-172	5.7	-2.6	0.8	-8.7	-20	-139	-173
2	0	4239	12	31	6	560	24.7	54.4	3.4	11	295	286	300	2	3.0	435	0.4	3.4	0.6	-8.9	-96	94	-56
3	15	1836	15	33	6	383	19.9	49.4	.8	11	380	284	340	1	2.0	-47	4.2	-3.6	0.1	-4.3	41	77	886
4	19	777	11	21	4	824	16.7	79.1	3.1	-3	323	364	223	4	2.8	285	7.3	17.7	0.4	-8.6	-38	-49	-13
5	5	2598	13	35	3	1034	50.7	100.6	1.6	12	574	463	855	1	1.1	-264	-6.7	-0.5	1.0	-2.9	-127	-91	-528
6	10	933	12	25	4	742	33.0	62.7	2.6	1	448	445	266	5	4.0	172	-0.8	-8.2	0.2	-2.3	-203	-151	208
7	13	2076	11	31	5	1125	27.5	67.3	-1.0	-4	319	476	318	5	4.7	-746	-1.9	1.5	3.6	21.3	70	-12	-20
8	9	499	10	23	3	576	40.2	100.7	.1	45	691	711	733	4	1.1	-219	12.1	0.1	0.4	-19.4	-233	-141	9
9	18	80	11	32	4	1026	42.1	80.7	2.2	5	308	390	306	4	0.7	-314	-11.4	-22.0	0.0	0.4	-73	9	-90
10	20	2730	10	39	5	1392	21.8	73.2	-.4	26	783	761	595	4	2.2	-900	14.3	-0.1	0.6	-8.7	-189	-21	-200
11	18	4530	12	33	2	694	33.3	47.6	3.7	15	283	235	174	6	5.9	-120	-14.9	-4.2	0.4	-0.2	-50	43	-9
12	10	1485	13	27	4	1429	28.7	81.0	4.3	27	491	416	322	5	6.2	-664	0.8	2.5	-2.6	8.9	-62	1	-29
13	24	1699	12	30	4	858	14.5	67.9	2.5	11	257	317	322	4	2.8	-344	0.2	6.5	2.0	-12.8	-41	-6	21
14	26	4164	11	35	4	202	37.7	74.1	3.1	1	378	323	274	6	8.8	127	-13.3	-12.4	0.5	1.1	-93	-41	-55
15	19	3375	10	33	3	332	18.1	70.5	.3	6	192	166	218	5	5.9	-19	2.7	-4.3	0.0	-7.2	-85	-16	-64
16	1	4640	11	22	8	828	37.3	41.0	-.2	0	534	474	376	4	1.8	-80	12.2	24.8	-0.2	17.6	-69	99	9
17	15	2937	12	29	3	442	20.2	90.8	2.2	19	240	230	149	3	4.4	-4	20.1	4.4	-0.1	-32.8	156	64	7
18	12	1017	19	36	8	694	47.1	98.8	-.3	30	414	428	192	4	1.0	-262	-6.9	-2.2	0.3	-27.6	-98	-53	18
19	25	4518	12	35	2	1341	24.6	101.6	.0	-2	149	111	100	2	3.4	-536	3.7	0.0	0.5	4.5	-33	-8	-14
20	24	5358	12	28	4	679	36.4	67.7	1.9	10	386	304	243	5	4.1	-286	-3.9	12.5	0.1	2.6	-150	-124	-112
21	11	600	11	31	4	329	17.5	71.5	2.4	2	680	483	590	1	1.0	46	-4.0	-11.1	-1.7	-6.4	-78	-66	-57
22	5	3970	13	34	4	335	24.7	68.8	-1.0	-1	273	303	220	3	1.7	167	4.4	0.6	1.8	-5.2	90	55	62
23	10	2946	12	22	3	1226	43.0	101.6	1.6	9	235	271	226	3	0.0	-317	-7.7	0.0	-0.2	-2.2	62	60	-12
24	19	1455	10	21	4	1068	32.0	71.7	-1.0	16	383	477	404	1	0.6	-37	-4.1	-20.7	2.2	-10.6	-119	-79	-128
25	16	1146	14	31	8	516	28.9	70.9	2.4	17	680	781	416	3	1.0	348	10.2	29.3	-1.3	6.5	12	-176	-95
26	8	4076	12	39	3	1308	31.7	101.5	-.7	-10	732	528	816	0	1.0	-131	3.8	0.1	1.4	-12.2	54	142	82
27	19	2826	12	23	7	1131	21.3	60.4	1.0	7	214	159	165	3	1.6	207	3.1	6.6	0.5	-3.6	22	21	-28
28	20	1200	11	31	2	520	20.3	41.5	-.2	-10	102	97	101	3	4.7	-129	-0.5	4.1	1.7	4.6	11	30	5

Note: ID, arbitrary number of subject; PCS, Pain Catastrophizing Scale; IPAQ, International Physical Activity Questionnaire (MET-min/week); Neg/Pos, Positive and Negative Affective Schedule – Negative/Positive subscales; PSQI, Pittsburgh Sleep Quality Index; Force, isometric force production (kPa); cPDT, cuff pain detection threshold (kPa); cPTT (cuff pain tolerance threshold); TSP, temporal summation of pain (cm); CPM, conditioned pain modulation (kPa); PPTi/c/u, pressure pain threshold (kPa) at index/contralateral leg/upper trapezius); Likert, scale of lower limb muscle soreness (0-6); VAS, visual analogue scale (0-10cm).
